# Photo-Induced Cycloaddition Reactions of *α*-Diketones and Transformations of the Photocycloadducts

**DOI:** 10.3390/molecules18032942

**Published:** 2013-03-04

**Authors:** Chengmei Huang, Mengmeng Zheng, Jianhua Xu, Yan Zhang

**Affiliations:** School of Chemistry and Chemical Engineering, Nanjing University, Nanjing 210093, China

**Keywords:** photocycloaddition, *α*-diketones, *N*-acetylisatin, phenanthrenequinone, isoquinolinetrione

## Abstract

Photocycloaddition, along with subsequent transformation of the photocycloadducts, provides expeditious ways to construct various structures. The photo-induced reactions of *α*-diketones have been reported to proceed via different reaction pathways with the involvement of one or two of the carbonyl groups. Photoinduced reactions of cyclic *α*-diketones including *N*-acetylisatin, phenanthrenequinone and isoquinolinetrione with different C=C containing compounds could take place via [2 + 2], [4 + 2] or [4 + 4] photocycloaddition pathways. We have investigated the photoreactions of these cyclic *α*-diketones with different types of alkenes and alkynes, with a focus on the unusual cascade reactions initiated by the photocycloaddition reactions of these cyclic *α*-diketones and the applications of these photocycloaddition reactions along with the transformation of the photocycloadducts. In this paper, we discuss the diverse photo-cycloaddition pathways found in the photocycloaddition of *o*-diones leading to various photocycloadducts and the potential applications of these reactions via further transformation reactions of the adducts.

## 1. Introduction

Photo-induced cycloaddition reactions combined with subsequent transformations of the photocycloadducts have played important roles in building diverse organic frameworks [1,2,3,4]. The application of photocycloaddition as the key step in the synthesis of natural products [5,6,7,8,9] and other novel frameworks is of current research interest [10,11,12,13,14,15]. The photo-induced [2 + 2] cycloaddition of carbonyl groups with C=C or C≡C groups, also known as the Paterno-Büchi reaction, is the most common photocycloaddition reactions of carbonyl compounds [16]. However, with the presence of an adjacent C=O, the carbonyl groups in *α*-diketones were found to have diverse cycloaddition pathways upon photo-initiation and various photocycloadducts other than oxetanes could be obtained. We have systematically investigated the photo-induced reactions of *α*-diketones including *N*-acetylisatin [17,18,19,20,21,22,23], 9,10-phenanthrenequinone [24,25] and 1,3,4-isoquinolinetrione [26,27,28] in the past decade. The photocycloadditions of these *α*-diketones, together with further transformations of the photo-cycloadducts have been demonstrated to be facile approaches to various polycyclic heterocycles which are otherwise hard to prepare.

In this review we will summarize the photocycloaddition pathways, including [2 + 2], [4 + 2] and [4 + 4] photocycloaddition, found in the reactions of *α*-diketones, with an emphasis on examples based on our previous work. Various compounds containing C=C or C≡C moieties could react readily with *α*-diketones upon photo-irradiation and the pathway partition in these photocycloadditions gave different cycloadducts or cascade products. The pathway partitioning depends not only on the nature of the *α*-diketones, but also on the substituents on the C=C or C≡C moieties. The photocycloadditions of *N*-acetylisatin with two adjacent carbonyl groups on the indole ring could proceed exclusively via [2 + 2] cycloaddition pathway with simple alkenes or via [4 + 4] cycloaddition pathway with specific oxazoles. Complex photocycloadducts have also been obtained from the [4 + 2] photocycloaddition initiated cascade reactions of N-acetylisatin. Competition of different photocycloaddition pathways has also been observed in the photoreactions of N-acetylisatin. The photocycloadditions of 9,10-phenanthrenequinone showed stronger competition of the [4 + 2] cycloaddition pathway to the [2 + 2] pathway. Besides, [4 + 4] cycloadducts were also found in the reactions of 9,10-phenanthrenequinone with oxazoles. In contrast, the two carbonyl groups in 1,3,4-isoquinolinetriones showed distinct reactivity in photocycloadditions. The C-4 carbonyl group is the common reactive site for photocycloaddition and the C-3 carbonyl group seldom gets involved in photoreactions, except in those with special alkenes such as bicyclopropylidene. Photo-induced cycloadditions of other *α*-diketones such as di-substituted *o*-benquinones and benzil via different pathways will also be introduced.

Transformations of photocycloadducts could provide facile ways to construct compounds with highly functionalized structures. Griesbeck *et al.* have reported that photocycloadditions of carbonyl compounds with oxazoles gave bicyclic oxetanes with highly regio- and diastereoselectivity and hydrolysis of the resulting adducts afforded *β*-hydroxy-*α*-amino acid derivatives. We found that hydrolytic cleavage of the bicyclic oxetanes formed by [2 + 2] cycloaddition between 1,3,4-isoquinolinetrione and 5-methoxyoxazoles went through a consecutive transformation to give isoquinolineoxazolines. Moreover, we have developed a facile method to prepare biaryl-fused bislactones using the reaction of [4 + 2] photocycloadducts of phenanthrenequinone with alkenes. The clean and efficient transformations of photocycloadducts from *α*-diketones will be reviewed in the third part of this paper.

## 2. Photo-Induced Cycloaddition Reactions of *α*-Diketones

### 2.1. *N*-Acetylisatin

1*H*-Indole-2,3-dione (isatin) derivatives have various biological activities [29]. They are basic structural units and important synthetic precursors of many natural alkaloids [30]. With acetylation on the nitrogen atom, *N*-acetylisatin (**IS**) becomes a better electron acceptor and shows much higher reactivity in photoreactions than the parent isatin. **IS** has been found to easily undergo photocycloaddition reactions with a wide range of alkenes [17,21,22], alkynes [18,23] and heterocyclic compounds [20,21,24], and hydrogen abstraction reactions with compounds such as aldehydes [19]. For the two adjacent carbonyl groups on the indole ring, the C3 carbonyl group in **IS** is more reactive than the C2 amide carbonyl group in photoreactions because the *α*-alkoxy carbonyl radical is more stable when it is at C3 rather than at C2 [19]. Therefore, all the photoreactions we investigated are initiated at the C3 carbonyl group. However, any subsequent reactions are highly dependent on the structures of the alkenes and may involve the C-3 carbonyl only ([2 + 2] reaction) or involve both C-3 and C-2 carbonyls ([4 + 2] and [4 + 4] reactions).

#### 2.1.1. [2 + 2] Photocycloadditions Involving **IS**

**Scheme 1:**
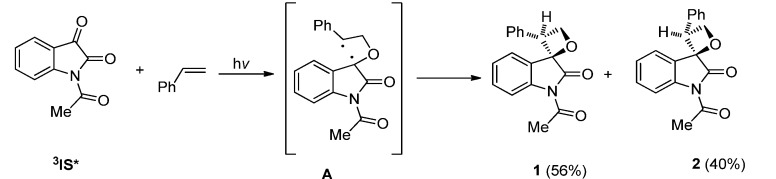
The photocycloaddition of **IS** and styrene.

Photoreactions of **IS** with ordinary alkenes or enol ethers are common Paterno-Büchi photocycloadditions with predictable regio- and diastereoselectivities. For example, the reaction of **IS** with styrene derivatives in benzene took place exclusively via the [2 + 2] pathway and gave the diastereoisomeric spiroxetane products via the nπ* triplet state of **IS** [22]. The regioselectivity and diastereoselectivity of the reactions depend on the reaction mechanism. In reactions with alkenes of high oxidation potential such as styrenes, the regioselectivity can be rationalized by the frontier molecular orbital (FMO) interactions of the excited **IS** with the ground state alkene and the formation of the most stable 1,4-diradical intermediate in the reaction. For example, the most stable benzyl 1,4-diradical intermediate **A** in the photoreaction of IS with styrene led to the formation of **1** and **2** (Scheme 1).

**Figure 1:**
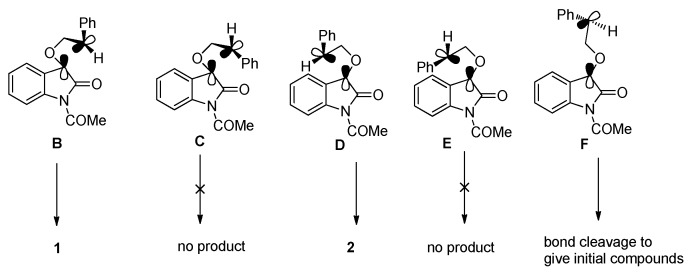
Possible projections of the1,4-diradicals formed in the photoreaction of **IS** with styrene.

The diastereoselectivity can be accounted for by the “Griesbeck model” [31] of the Salem-Rowland rules [32,33,34,35,36] for intersystem crossing (ISC). Before the intermediary triplet 1,4-diradicals transit to closed-shell products, they must go through ISC to realize spin inversion. The rate of ISC is controlled by spin orbit coupling which depends on the geometries of the diradicals. For the sake of the most effective ISC and C-C bond formation, the axes of the *p*-orbit of the diradical should be vertical. There are four possible projections leading to *trans*- or *cis*- products formation (Figure 1 **B**–**E**), while there are also other conformers (such as **F**) which are suitable for efficient ISC but are not suitable for bond formation because of the long distance between the two radical centers. As for the conformers **C** and **E**, there is strong steric hindrance between the phenyl and the carbonyl or phenyl of the isatin framework. Without steric hindrance between phenyl and the isatin framework, **B** and **D** are favorable conformers. Compared with **B**, there is a little steric hindrance in **D** between the hydrogen atom and the isatin ring. Therefore the formation of the *syn*-oxetane cycloadduct via **B** is the most favorable. For the more electron-rich alkenes such as stilbenes with electron-donating substituents, single electron transfer (SET) processes with ^3^IS* and ion-radical pair formation are energetically feasible. The regioselectivity of the photocycloaddition is dependent on the charge and spin-density distribution in the ion-radicals [22].

**Scheme 2:**
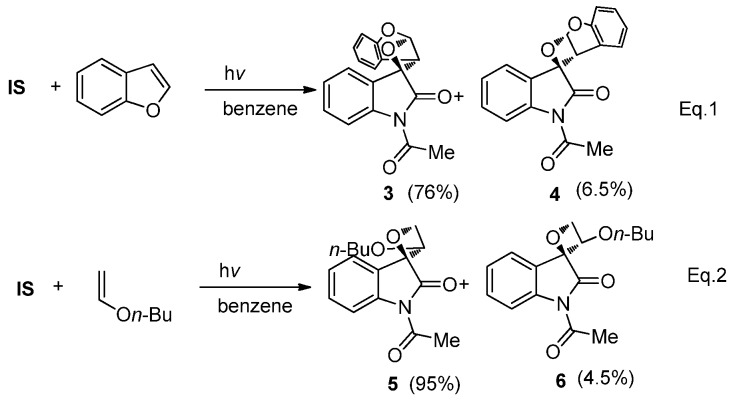
The photoreactions of **IS** with enol ethers.

Photoreactions of **IS** with cyclic or acyclic enol ethers also afforded the bicyclic spiroindoleoxetanes in high yields with high regio- and diastereoselectivity [21]. For example, irradiation of a benzene solution containing **IS** and benzofuran (λ > 400 nm) gave the two head-to-head isomers **3** and **4** with a ratio of 12:1 (Equation 1 in Scheme 2). Similar reaction between **IS** and *n*-butyl vinyl ether afforded head-to-tail product **5** and **6** with preference for the product **5** with *syn*- configuration (Equation 2 in Scheme 2).

**Figure 2:**
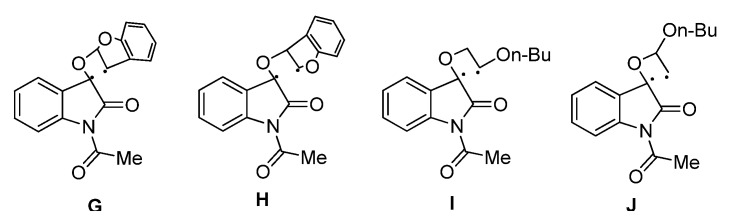
Different 1,4-diradical conformers.

The regio- and stereoselectivity can be explained by the classical 1,4-triplet biradical mechanism without SET involvement. In the photocycloaddition of **IS** with cyclic enol ethers like benzofuran, the 1,4-diradical intermediate **G** with a benzyl radical is much more stable than the *α*-oxygen substituted 1,4-diradical **H** (Figure 2). Therefore the head-to-head photocycloadducts **3** and **4** were found to be the predominant regioisomers. Meanwhile, a charge density calculation has shown that in the vinyl alkyl ether cation radical, the two vinyl carbon atoms have comparable positive charge density [37], which indicates the 1,4-diradical **I** with an electon donor is much more stable than **J** (Figure 2). Therefore the formation of head-to-tail cycloadducts **5** and **6** via **I** was found to be predominant. The sterically more favorable 1,4-diradical conformers suitable for the efficient ISC and C-C bond formation resulted in the thermodynamically less stable *syn-*spirooxetanes as the main diastereoisomer.

**Figure 3:**
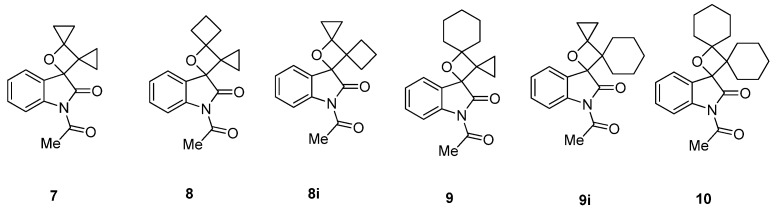
Spirooxetanes derived from [2 + 2] photocycloaddition of **IS** and bicycloalkylidenes.

Except for the conventional photoreactions of **IS** with ordinary alkenes, enol ethers and enol ester, we have also investigated the uncommon photoreactions of **IS** with strained alkenes and special C=C containing species such as oxazoles. In these photoreactions, the [2 + 2] cycloadducts were formed in competition with other products via different light-initiated pathways which will be reviewed later in this paper. For example, we reported the photoreactions between **IS** and bicycloalkylidenes including bicyclopropylidene (**BCP**), cyclopropylidenecyclobutane (**CPCB**), cyclopropylidenecyclohexane (**CPCH**) and bicyclohexylidene (**BCH**) [17]. In these unconventional alkene species with unusual bonding properties [38,39,40,41], the extra strain led to unusual reactivities in their photoreactions with **IS**. Although spirooxetanes **7**–**10** derived from the [2 + 2] cycloaddition pathway can be isolated from the reaction mixture (Figure 3), their overall yields just account for less than 40% of the main products. It is noteworthy that the photoreactions of **IS** with asymmetric bicycloalkylidenes like **CPCB** or **CPCH** gave the regioisomers **8**/**8i** and **9**/**9i** respectively. The formation of **8** or **9** was found to be preferred over that of **8i** and **9i**, which might be explained by the fact that the steric hindrance for the 1,4-diradical recombination in the former with cyclopropane is less than that in the latter with cyclobutane or cyclohexane.

**Scheme 3:**
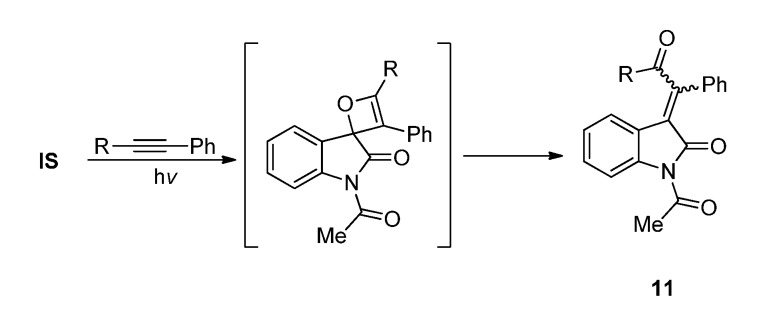
The photoreactions of **IS** with 1,2-disubstituted acetylene.

Photocycloadditions of carbonyl compounds with alkynes have not been as extensively explored as those with alkenes. It is known that the Paterno-Büchi reactions of ordinary carbonyl compounds with alkynes usually give *α,β*-unsaturated carbonyl compounds (the quinone methides) as a result of the spontaneous rearrangements of the thermally labile oxetenes formed by the [2 + 2] cycloaddition [42,43,44,45,46,47,48,49,50]. The electron-withdrawing acetyl group enables **IS** to react with a variety of alkynes including electron deficient terminal alkynes such as phenylacetylenes or cyclopropylacetylene [18,23] which seldom took part in photocycloadditions with other carbonyl compounds. Photoreaction of **IS** with 1,2-disubstituted acetylenes also proceeded via the [2 + 2] cycloaddition pathway followed oxetene ring opening to give the *β,β*-disubstituted 3-alkylideneoxindoles **11** (Scheme 3) [18,23].

**Scheme 4:**
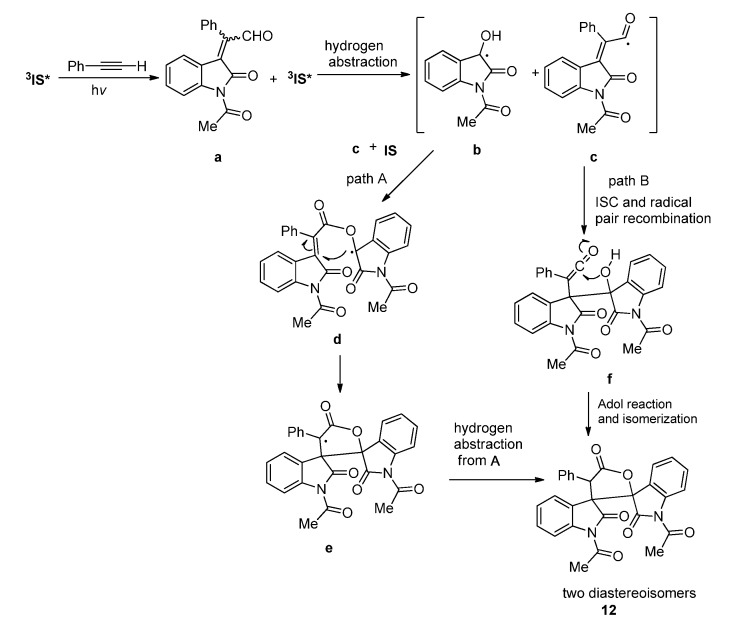
The photoreactions of **IS** with phenylacetylene.

On the other hand, photoreactions of **IS** with terminal alkynes such as phenylacetylenes were found to give complex polycyclic products like the dispiroindole[3,2']furan [3',3'']indole derivatives **12** via a complex tandem reaction course [18,19,23] (Scheme 4). We have investigated the mechanism of this cascade reaction. Firstly, triplet excited **IS** reacted with phenylacetylene to form quinone methide **a**. Intermediates **b** and **c** were produced via hydrogen abstraction between **a** and another triplet excited **IS**. In pathway A, an oxygenphilic attack of the carbonyl radical furnished radical **d**. An intramolecular radical cyclization in **d** followed by hydrogen abstraction results in the formation of **12**. Alternatively, radical pair **b** and **c** may undergo an in-cage radical pair recombination after ISC to give the ketene product **f** (pathway 2 in Scheme 4).Aldol reaction and isomerization of **f** gave products **12**.

#### 2.1.2. Photoreaction of **IS** Initiated by [4 + 2] Cycloaddition

With the two adjacent carbonyl groups, **IS** could also serve as 4*π* addend to take part in the higher ordered cycloadditions. The 1,4-diradical intermediate formed between the nπ* triplet excited **IS** and the alkene has a delocalized spin density, which allows the presence of the 1,6-diradical intermediate. The [4 + 2] cycloadducts of **IS** with alkenes are usually highly reactive and can react rapidly with another C=O or subject to other fast transformations. In the photoreaction of **IS** with bicyclohexylidene under inert atmosphere, tiny amount of the [4 + 2] cycloadduct was isolated [17]. Although the yield of the [4 + 2] cycloadduct was as low as 2%, it did provide the direct evidence for the [4 + 2] cycloaddition pathway involved in the photoreaction of **IS** with strained alkenes.

**Scheme 5:**
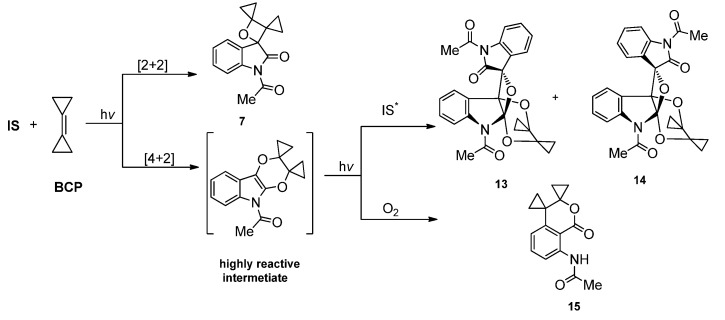
The photoreactions of **IS** and **BCP**.

In the photoreaction of **IS** with bicyclopropylidene which is highly strained, the [4 + 2] cycloaddition pathway was involved in the formation of two type of final products via the [4 + 2] cycloadduct as intermediate (Scheme 5) [17]. When the reaction was conducted under an inert atmosphere with the least amount of oxygen, the highly reactive C=C in the [4 + 2] photocycloadduct could react with another excited IS via [2 + 2] pathway to give the final [4 + 2 + 2] cycloadducts, which could be obtained as two diastereoisomer **13**/**14** with a total yield of 10%. Meanwhile, the highly reactive [4 + 2] intermediate could also be photooxygenated under the action of the trace amount of oxygen in the reaction system to afford the oxoisochroman **15** (Scheme 5). It is observed that the formation of **13**/**14** and **15** seemed to be competitive to each other and a higher concentration of dissolved O_2_ favored the formation of **15** [17].

#### 2.1.3. [4 + 4] Photocycloaddition of **IS** with Oxazoles

Photoinduced [4 + 4] reactions represent the highest order photocycoadditions of α-diketone known up to now. [4 + 4] Photocycloadditions have been found in some photoreactions of excited aromatic compounds with dienes [51,52,53,54,55,56,57]. In an isolated example, excited 1,2-naphthoquinone was reported to react with cycloheptatriene to give the [4 + 4] product as minor products [58]. In the photoreactions of **IS** with some oxazoles, we observed the involvement of [4 + 4] cycloaddition pathway in the formation of complex photocylcoadducts.

**Scheme 6:**
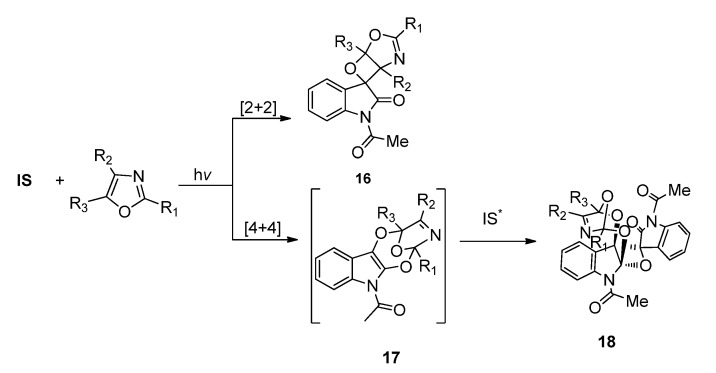
The photoreactions of **IS** and oxazoles.

As shown in Scheme 6, except for the common [2 + 2] photocycloaddition leading to the spirooxetanes, [4 + 4] cycloaddition of the O=C-C=O in **IS** and the C=C-C=N in oxazoles also happened when the oxazole was properly substituted. Substitution groups on the oxazole showed great influence on the preference of the [2 + 2] or [4 + 4] cycloaddition pathway. For example, the 2,4,5-trimethyloxazole reacted with **IS** via [2 + 2] cycloaddition pathway exclusively. However, in the reaction of **IS** with 4-phenyloxazole, the product derived from the [4 + 4] cycloaddition initiated pathway could be isolated with yield up to 97% [24]. The [4 + 4] photocycloadduct was also highly reactive and could react rapidly with another excited IS to give the final [4 + 4]/[2 + 2] product **18**.

**Scheme 7:**
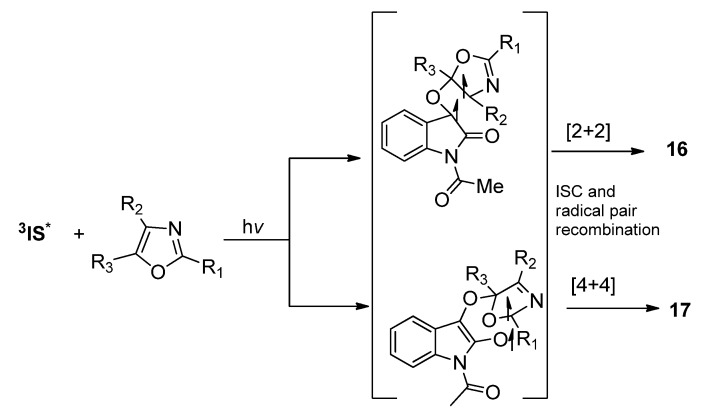
The chemo-selectivity in the photoreaction of **IS** and oxazoles.

The reason for the dependence of chemoselectivity on the substitution groups on the oxazole ring has been rationalized. The steric hindrance from R_1_ group hampers the radical pair recombination in the corresponding 1,7-diradical to give the [4 + 4] cycloaddition intermediate **17** (Scheme 7), while the R_2_ group may cause steric hindrance to the 1,4-diradical recombination and therefore disfavors the [2 + 2] cycloaddition to form **16**. In accord with this, the 4-aryloxazoles without a substituent R_3_ at the C5 atom in the oxazole ring give the [4 + 4] products **18** exclusively. The regio- and diastereoselectivity can also be explained by consideration of the most stable diradicals and the most favorable diradical conformation for the ISC and subsequent C-C bond formation events.

We have also investigated the photoreactions of **IS** with other aza-aryls such as imidazole and thiazole. Compared with oxazoles, both imidazole and thiazoles had much lower reactivity in the photoreaction with **IS**. The photocycloadducts obtained in these reactions were derived from the [2 + 2] cycloaddition pathway. Our exploration in the photoreactions of **IS** has demonstrated that **IS** is a special *α*-diketone species with diverse transformation pathways in photo-initiated cascade reactions. We are still putting efforts to discover more and more **IS**-involved photoreactions and found their applications in the synthesis of compounds with biological activities.

### 2.2. Phenanthrenequinone

The photocycloaddition reactions of 9,10-phenanthrenequinone (**PQ**) as another common *α*-diketone have been studied by us and others. The photoreactions of **PQ** with olefins have been known for a long time and its synthetic applications and mechanism have been studied in detail for decades [59,60,61,62]. The photocyloaddition of **PQ** with C=C usually leads to two types of photocyloadducts, that is, the [2 + 2] keto oxetanes derived from [2 + 2] cycloaddition and the dihydrodioxines from the [4 + 2] cyclo-addition pathway. We will briefly review some of the photocycloadditions of **PQ** with high chemoselectivity, together with the unusual photocycloadditions happened via [4 + 4] pathway.

#### 2.2.1. [2 + 2] Photocycloaddition of **PQ**

**Scheme 8:**
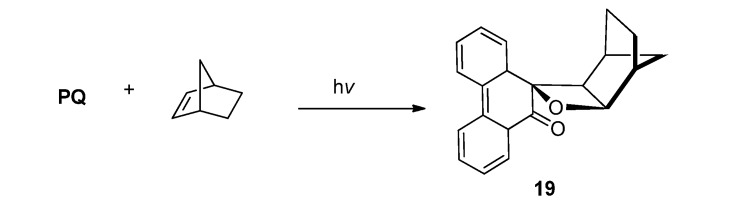
The photoreaction of **PQ** and bicyclic olefin.

Photocycloaddition reactions of **PQ** with alicyclic olefins and bicyclic olefins upon irradiation with visible light (>420 nm) was examined by Maruyama *et al*. [59]. They found that the reaction of **PQ** with bicyclic olefins exclusively gave keto oxetanes via the [2 + 2] photocycloaddition pathway (Scheme 8). Other alicyclic olefins gave not only keto oxetanes but also dihydrodioxines and photoreducts. The differences of the chemoselectivity were elucidated in terms of the character of intermediary biradicals.

#### 2.2.2. [4 + 2] Photocycloaddition of **PQ**

**Scheme 9:**
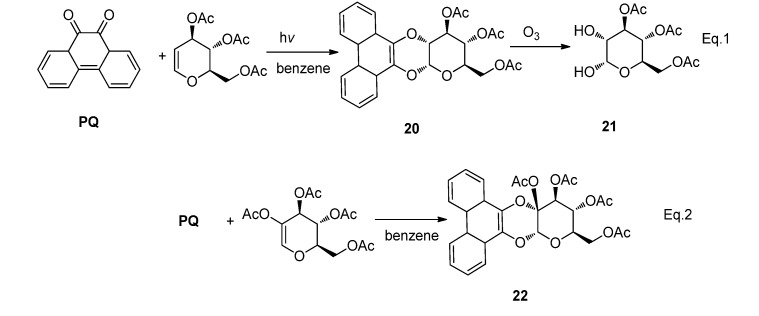
The photoreaction between **PQ** and 3,4,6-tri-*O*-acetyl-D-glucal or 2-hydroxyl glucal esters.

The most widely reported photo-induced reactions between **PQ**s and alkenes are the [4 + 2] photocycloadditions. Early in 1950s, Helferich and co-workers have already performed the UV-promoted cycloaddition of **PQ** to tri-*O*-acetyl-D-glucal to give the [4 + 2] cycloaddition product **20** in 50% yield. It is noteworthy that **20** has a phenanthrodioxenopyran with an *α*-D-gluco-configuration at the pyran position and the reaction has been used to further prepare 3,4,6-tri-*O*-acetyl-D-glucose **21** through ozonolysis of **20** (Scheme 9) [63]. Later it was reported that other 2-hydroxyglucal esters similarly reacted with phenanthrenequinone to give the [4 + 2] cycloadduct **22** with high chemoselectivity [64]. Photocycloadditions of acenaphthenequinone with tri-*O*-acetyl-D-glucal exclusively gave the [2 + 2] cycloadduct. In our recent work, using the [4 + 2] photocycloaddition to generate dihydrodioxines for further photooxidation, we found that irradiation of PQ with strained alkenes such as BCP or BCH in benzene gave the [4 + 2] photocycloadducts with 80–90% yields [25]. A direct attack of the ketone diradical to the C=C bond leading to the 1,6-diradical intermediate was considered to be a preferred model of the [4 + 2] photocycloaddition [65].

#### 2.2.3. [4 + 4] Photocycloaddition of **PQ**

**Scheme 10:**
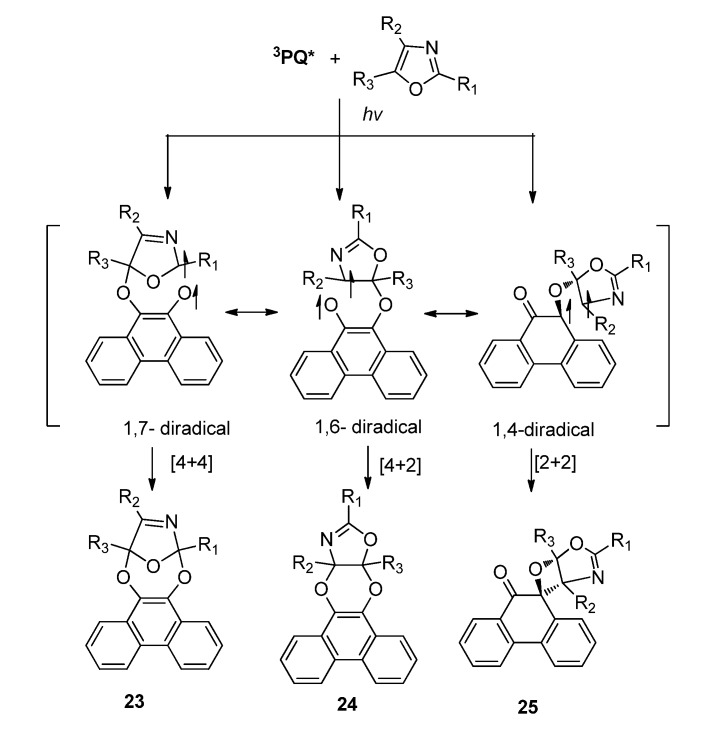
The photoreactions of **PQ** and oxazoles.

The *α*-dicarbonyl functionality (O=C-C=O) in **PQ** could also serve as *4π* addend to react with dienes or heterodienes via [4 + 4] cycloaddition [20,24]. In our group, we first reported the [4 + 4] cycloaddition between two heterodienes. Photocycloadditions of **PQ** and oxazoles gave [4 + 4] products 23 with the 2-azadiene moiety in oxazole as another 4π addend, along with the [4 + 2] cycloadduct 24 and the [2 + 2] cycloadducts **25** (Scheme 10). As we discussed before, the chemo-selectivity in the photoreactions between **PQ** and oxazoles depend highly on the substitution groups on the oxazoles. The effect of substituents on the oxazole ring on the pathway partitioning in the photocycloaddition of **PQ** with these oxazoles showed slight differences from that of **IS** with oxazoles. For example, the reaction of **PQ** with 2,4,5-trimethyloxazole gave the [4 + 4] photocycloadduct with yields up to 89%. Besides, the [4 + 4] photocycloadduct **23** did not undergo further photocycloaddition.

### 2.3. Isoquinoline-1,3,4-trione

Isoquinolinetriones (**IQT**) are important biologically active compounds [66,67] and they have been used as building blocks in the synthesis of benzo[c]phenanthridine alkaloids [68,69]. Photoreactivity of **IQT** was not as high as that of **IS** or **PQ** since they could barely react with common alkenes upon photo-irradiation. It also took longer for the total conversion of **IQT** in their photoreactions with acetylenes or with special C=C containing species such as oxazoles or strained alkenes. In most of these photoreactions of **IQT**, the C4 carbonyl group on the isoquinoline ring was found to be the only reactive site.

#### 2.3.1. Photoreaction with C=C Containing Species

**Scheme 11:**
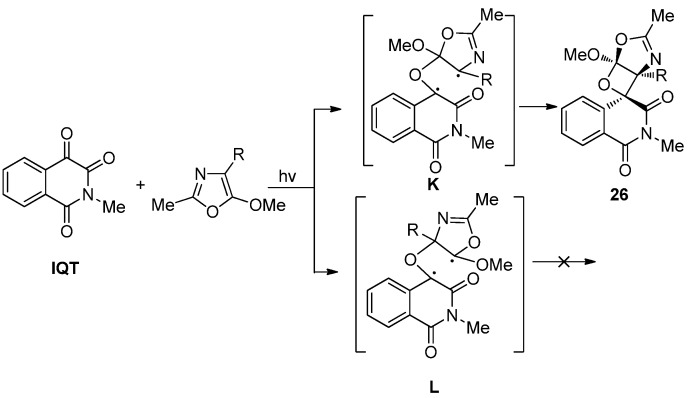
The photocycloaddition of **IQT** with 2-methyl-5-methoxyoxazoles.

We have studied the photoreactions between **IQT** and 2-methyl-5-methoxyoxazoles [27]. The reactions were found to proceed exclusively via a [2 + 2] cycloaddition pathway with excellent chemo-, regio- and diastereoselectivity. The chemoselectivity was much higher than that found in photoreactions of other *o*-quinones such as **IS** and **PQ** [20,24]. The resulting spiroisoquinolineoxetanes **26** were produced with *exo-*configuration exclusively (Scheme 11). The regioselectivity of the photocycloaddition is determined by the most stable 1,4-diradical intermediate. As shown in Scheme 11, two possible biradical intermediates **K** and **L** could be generated in the photocycloaddition of **IQT** with 2-methyl-5-methoxyoxazoles. **K** is an allylic type radical while **L** only has an oxygen *α* to the radical center. Therefore the [2 + 2] cycloaddition proceeded exclusively via **K** to give the oxetane **26**.

**Figure 4:**
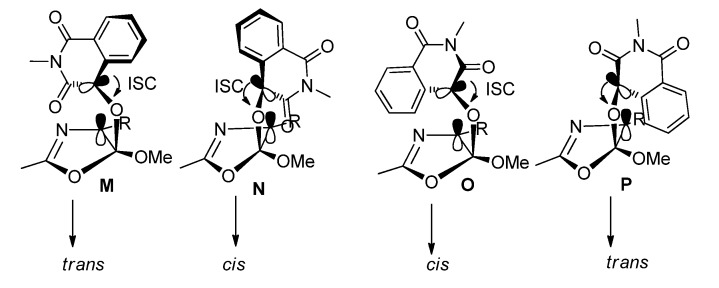
Possible projections in the formation of the [2 + 2] photocycloadducts of **IQT** with 2-methyl-5-methoxyoxazoles.

The diastereoselectivity can also be explained by the Salem-Rowland rule. There are four possible projections leading to *trans*- or *cis*- products formation (Figure 4). There is steric hinderance between C3 carbonyl group of **IQT** and the R group (**N**) or between the benzene ring of **IQT** and the R group (**P**). The steric hinderance makes the ISC process require much more energy. There is some hinderance between the benzene ring of **IQT** and the oxazole ring (**O**), while the steric hinderance in **M** is the weakest, which makes the yield of the *trans*- product be higher than that of the *cis*-product.

**Scheme 12:**
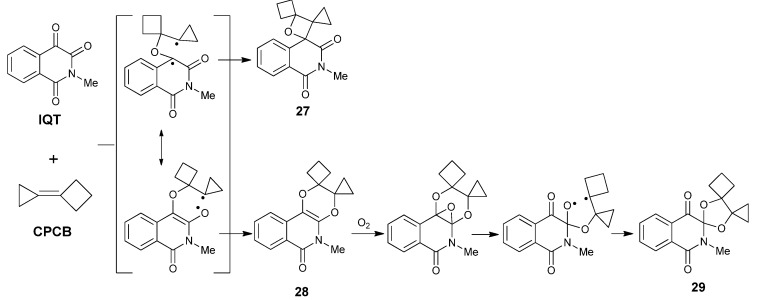
The photoreaction between **IQT** and cyclopropylidenecyclobutane.

By far, the only higher order photoreaction of **IQT** in which both the C3 and the C4 carbonyl groups were involved is the reaction between **IQT** and the highly strained bicycloalkylidenes. We found that in photo-induced reaction of **IQT** with **CPCB** in acetonitrile, three types of products were formed (Scheme 12). Except for the spirooxatane **27** from the [2 + 2] cycloaddition pathway, we also isolated small amounts of the [4 + 2] cycloadduct **28**, which is thermally unstable and can be easily converted into **29** upon photoirridiation in the presence of O_2_ [17].

#### 2.3.2. Photoreactions with Acetylenes

**Scheme 13:**
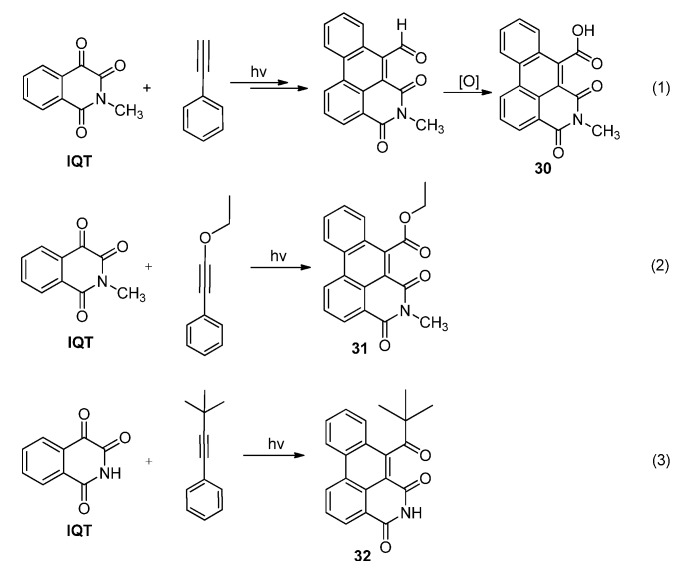
Photoreactions of **IQT** with differently substituted phenylacetylenes.

We have systematically studied the photoreactions of **IQT** with various acetylenes [26,28]. Compared with **IS**, **IQT** showed much lower reactivity in its photoreaction with acetylenes. Therefore photo-irradiation for much longer times was needed to achieve modest conversion of **IQT** if the acetylene was not substituted by electron-rich groups. For example, in the photoreaction of **IQT** with phenylacetylene (Equation 1 in Scheme 13), the conversion of **IQT** was less than 20%, even after 72 hours’ irradiation. Electron-rich substitution group linked to the C≡C could greatly accelerate the cascade reaction. Photo-irradiation of **IQT** with ethoxyphenylacetylene or *t*-butylphenylacetylene for less than 12 hours could lead to complete conversion of **IQT** and the formation of the polycyclic products **31** and **32**, respectively, in high yields (Equations 2 and 3 in Scheme 13).

**Scheme 14:**
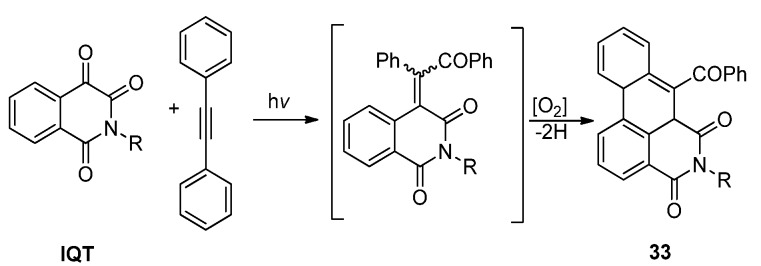
The photoreaction between **IQT**s and diphenylacetylene.

**Scheme 15:**
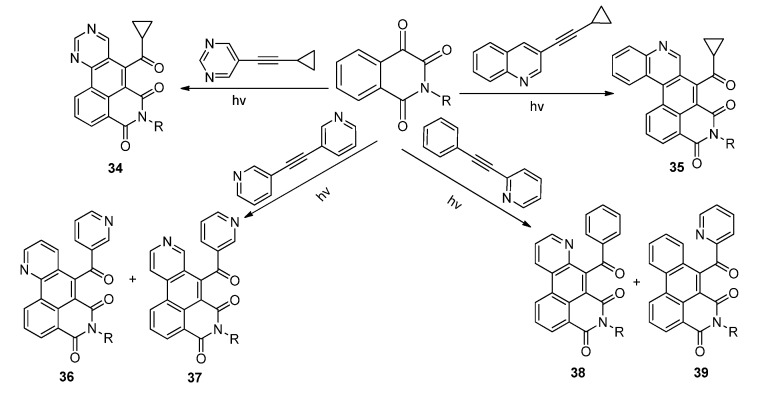
The photoreaction between **IQT**s and aryl acetylenes

In the photoinduced reactions of **IQT** with diphenylacetylene in acetonitrile, we found that the reaction proceeded smoothly and the product, which precipitated readily from the reaction mixture, was later confirmed to be the dibenz[*de,g*]-isoquinoline-4,6-dione derivatives **33**. The reaction was proposed to proceed via a sequential [2 + 2] photocycloaddition, ring rearrangement of the oxetene and dehydrogenative cyclization, as shown in Scheme 14 [28]. Using the photo-tandem reactions of isoquinolinetrione with acetylenes substituted by different azaaryl rings including pyridine, pyrimidine, pyrazine, and quinoline, we were able to obtain diverse aza-polycyclic frameworks **34**–**39** with isoquinolinedione fused with naphthalene, quinoline or isoquinoline, quinazoline, and phenanthridine respectively, in yields up to 85% (Scheme 15).

### 2.4. Photocycloadditions of Other *α*-Diketones

#### 2.4.1. Cyclic *α*-Diketones

**Scheme 16:**
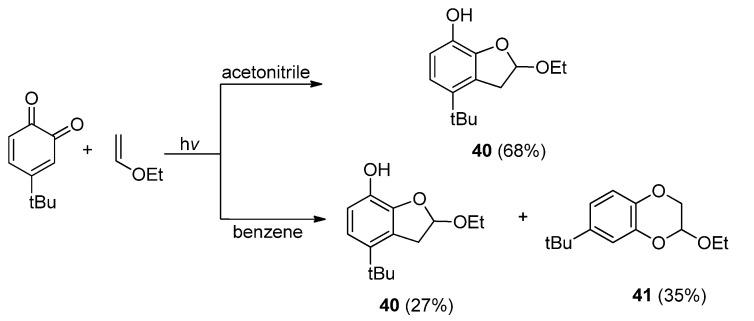
The photocycloaddition between *o*-benzoquinone and ethyl vinyl ether.

There are several other types of cyclic *α*-diketones reported to react with C=C or C≡C compounds via photocycloaddition pathways. The photocycloadditions of 1,2-naphthoquinone with simple olefins such as styrene were reported to only give [4 + 2] cycloadducts [70], while in the photoreactions of *o*-benzoquinones, including 1,2-naphthoquinone, with electron-rich olefins such as vinyl ethers [71,72,73] and allylsilanes [10,11,12,13,14,15,74], [3 + 2] photocycloadducts with dihydrobenzofuran frameworks could also be formed in polar solvents. For example, irradiation of *o*-benzoquinone with ethyl vinyl ether in acetonitrile with light of λ > 450 nm gave dihydrobenzofuran **40** via regioselecive [3 + 2] photocycloaddition, whereas in benzene, the same photoreaction gave [4 + 2] cycloadducts **41** along with **40** via a competitive cycloaddition pathway (Scheme 16). The pathway partition depends not only on the substituent groups on the benzoquinone, but also on the solvents used for the photoreactions [71,72].

**Scheme 17:**
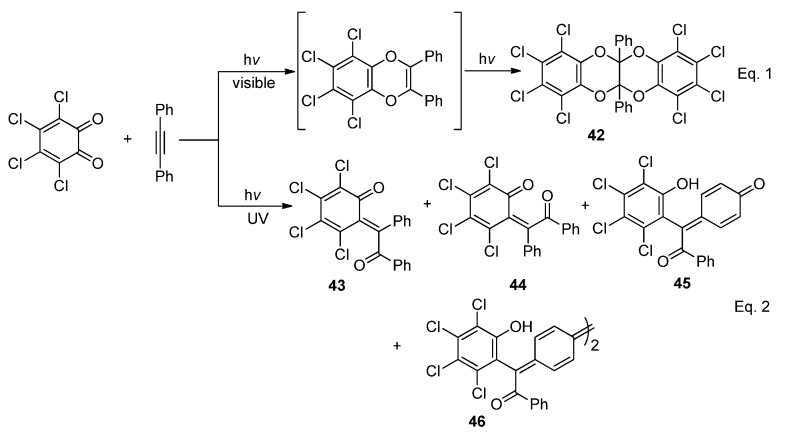
The photoreaction of tetrachloro-1,2-benzoquinone with diphenylacetylene.

The photoreactions of the symmetric *α*-diketones such as tetrachloro-1,2-benzoquinone with acetylenes have been reported to be highly dependent on the light source. Photoreaction of tetrachloro-1,2-benzoquinone with diphenylacetylene in acetone or acetonitrile irradiated with light of wavelength longer than 400 nm gave the 1:2 adduct **42** via a [4 + 2] cycloaddition pathway (Scheme 17 Equation 1) [75], while two isomeric *o*-quinomethanes **43** (*E*-) and **44** (*Z*-) were formed when *o*-quinones were irradiated with diphenylacetylene in dichloromethane under 300 nm UV light. Irradiated at 300 nm, the *E-*isomers could isomerize to the *Z-* form, but the *Z*- isomer could not. Novel *p*-quinomethanes **45** and quinodimethanes **46** (at high concentration) were also formed through the initially produced *o*-quinomethanes with molecular oxygen (Scheme 17 Equation 2) [49,76].

Benzo[*b*]thiophene-2,3-dione is also known as thioisatin. Its photoreaction with alkenes has been reported to proceed exclusively via the [4 + 2] cycloaddition pathway to give the 1,4-dioxin type products [77]. Acenaphthenequinone has been compared with phenanthrenequinone in their photocycloadditions with 3,4,6-tri-*O*-acetyl-D-glucal. While phenanthrenequinone preferentially adds with both carbonyl oxygens to give the [4 + 2] cycloadduct, acenaphthenequinone reacted with only one carbonyl group to exclusively yield the [2 + 2] addition product [64]. In the photoreactions of acenaphthenequinone with silyl ketene acetals in benzene, the [4 + 2] photocycloadducts could be obtained with modest yield [78].

#### 2.4.2. Acyclic *α*-Diketones

Photocycloadditions of acyclic *o*-diones including biacetyl, benzil and 1,2-diketones conjugated with ene-yne have been reported. The photocycloaddition of biacetyl with olefins such as indene, furan, and enol ether gave oxetanes with higher orientational selectivity than in the case of monoketones [78,79]. The photoreaction of biacetyl with electron-deficient olefins did not give cycloadducts, indicating that the excited biacetyl was electrophilic in its reaction with olefins. Besides, the phosphorescence of biacetyl could be quenched by an olefin, indicating that the nπ* triplet state of biacetyl was involved in these reactions. Therefore the reaction mechanism was proposed to be an electrophilic attack of the nπ* triplet state of biacetyl on the olefins to give a biradical intermediate.

It has been reported that photoirradiation of benzil promotes homolytic cleavage of the central C-C bond to generate benzoyl radicals and lead to products like benzoic acid and benzaldehyde [80]. In our work using benzil as the *α*-diketone to react with oxazoles, [2 + 2] photocycloadducts were obtained as the exclusive photocycloadducts [24]. However, in a recent work using benzil as the 1,2-diketone to react with silyl ketene acetals in benzene solution, production of the [4 + 2] cycloadduct 1,4-dioxene was observed [78].

**Scheme 18:**
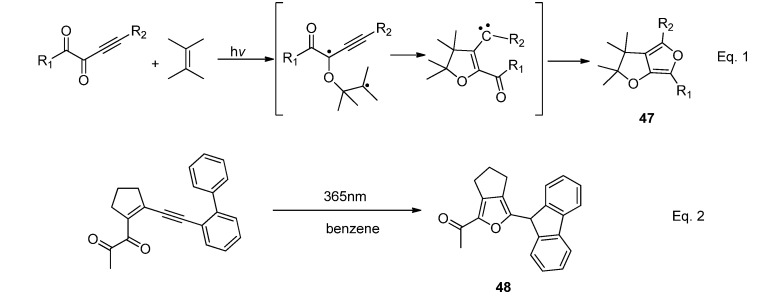
The photoreactions of conjugated *α*-diketones.

Photocycloaddition of conjugated *α*-diketones with alkenes has been found to be a clean approach to tetrasubstituted furans (Scheme 18, Equation 1). In the proposed mechanism, the nπ* excited triplet state of the *α*-diketones conjugated with C≡C reacted with alkene to furnish an alkyl propargyl biradical, which further underwent 1,5-closure to give a vinyl carbene. Trapping of the carbene in nonprotic solvents by the adjacent carbonyl group led to the formation of the 2,2'-bifuran derivative **47**. The photocyclization of 1,2-diketones conjugated with ene-yne moieties to (2-furyl)carbene was employed as a carbene-generating system. For example, in aprotic solvents the photo-irradiation of the ene-yne conjugated 1,2-diketones which bear a biphenyl system as a carbene trapping unit could initiate tandem cyclizations via a carbene intermediate. The reaction led to the formation of fluorenylfuran derivative **48** in nearly quantitative yield (Scheme 18, Equation 2) [81].

## 3. Transformations of Photocycloadducts

The photocycloadducts of *α*-diketones can be highly reactive and subject to sequential transformations under the same photoreaction conditions. As mentioned above, the [4 + 2] photocycloadducts of **IS** or **IQT** with strained alkenes could barely be isolated from the photoreaction mixtures. Transformation of this type of highly reactive photocycloadducts could not be controlled and applied in synthetic chemistry. However, there are moderately reactive photocycloadducts of *α*-diketones with various alkenes. These photocycloadducts are stable enough to be isolated and purified from the photoreaction mixture. Their transformation under specific conditions thus became controllable and could be developed into useful methods to construct special structural motifs. In this part we will summarize our efforts to develop useful synthetic protocols based on transformations of photocycloadducts of *α*-diketones.

### 3.1. Transformations of Oxetanes

**Scheme 19:**
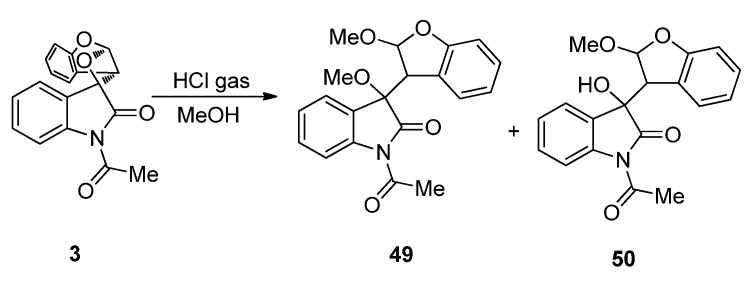
The acid-mediated transformation of spiroindoleoxetane **3**.

The [2 + 2] photocycloadditions of cyclic *α*-diketones with alkenes provide a convenient access to spirooxetanes. The oxetanes are usually acid- or thermolabile products. It is reported that the oxetanes derived from the photoreactions between the carbonyl group in aldehydes or ketones and the double bond in heterocyclic rings such as furans or oxazoles could transform into the corresponding *β*-hydroxy ketones [82,83,84,85,86,87,88,89]. Griesbeck *et al*. have developed convenient methods to prepare α-amino-β-hydroxy carboxylic acid derivatives through acid catalyzed hydrolysis of oxetanes derived from photocycloaddition of 5-alkoxyoxazoles with aldehydes [90,91]. The oxetanes derived from *α*-diketones and alkenes can also undergo acid-mediated transformation reactions. For example, purging dry HCl gas into a solution of **3** derived gave two ring cleavage products **49** and **50** (Scheme 19) and can serve as an efficient approach to indole derivatization at C(3).

**Scheme 20:**
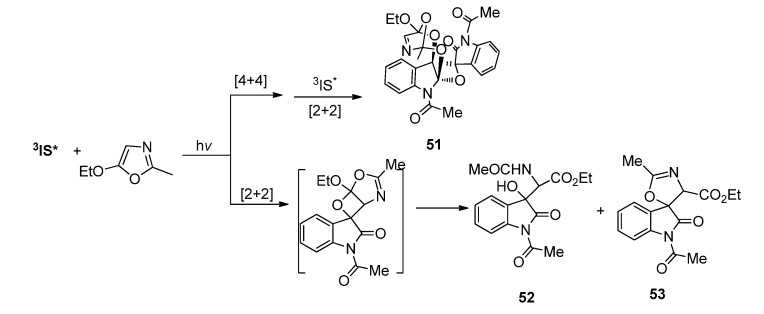
The transformation of spiroindoleoxetane derived from **IS** and 2-methyl-5-ethoxyoxazole.

In our work using 2-methyl-5-ethoxyoxazole to react with **IS** under photoirridiation, we observed the formation of two secondary products **52** (31%) and **53** (22%) along with the [4 + 4]/[2 + 2] adduct **51** (12%). The formation of **52** was due to the lability of the corresponding oxetane product in the work-up process under mild acidic conditions, which was similar to that reported by Griesbeck *et al*. However, the formation of the spirooxazoline product **53** was unusual since it involved the intramolecular cyclization of **52** (Scheme 20) [24]. We then proposed that the acid-mediated transformation of spirooxetanes might involve cascade reactions leading to novel organic frameworks. Therefore we prepared a series of spirooxetanes through the highly regio- and diastereo-selective photocycloaddition of **IQT** with 2-methyl-5-methoxyoxazoles. The acid-mediated transformation of these spirooxetanes under different acid condition was studied in detail.

**Scheme 21:**
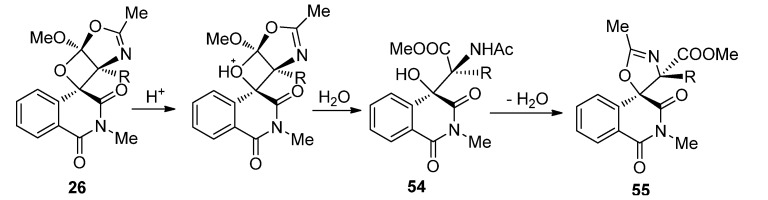
The sequential transformation of **26** under acid catalysis.

We found that acid-catalyzed transformation of the spiroisoquinolineoxetanes was highly related to the substituent groups on the oxetane rings as well as the type and amount of acid used. Novel spiroisoquinolineoxazoline products **55** could be obtained upon treatment of the spiroisoquinolineoxetanes with large excess amounts of concentrated HCl or with catalytic amounts of strong organic acids like TFA, MsOH or strong Lewis acids like BF_3_·Et_2_O and TiCl_4_ (Scheme 21) [27]. Bulky substituent groups on the oxetane ring showed favorable steric effects on the transformation from **26** to spiroisoquinolineoxazolines **55**. When R was H in **26**, the consecutive transformation from **54** to **55** did not happen under mild acidic conditions, but when R was an isopropyl group in **26**, the transformation could not stop upon generation of the **54** type product and the spiroisoquinoline-oxazoline type product **55** was the only product obtained. Large excess amount of HCl also favored transformation from **26** to **55**. Strong Brönsted acids and Lewis acids could catalyze direct transformation from **26** to **55** efficiently, but via different pathways leading to different ratios of diastereoisomers.

We proposed two reaction pathways to explain the acid-mediated transformation. In pathway A, there was no stereogenic center involved, so the configuration was conserved. In pathway B, the C–O bond between the isoquinoline and the oxetane O underwent bond cleavage, leading to the carbocation as an intermediate. Nucleophilic attack of the carbonyl group to the carbocation could proceed from different directions to give **55** and **55i**, respectively.

**Scheme 22:**
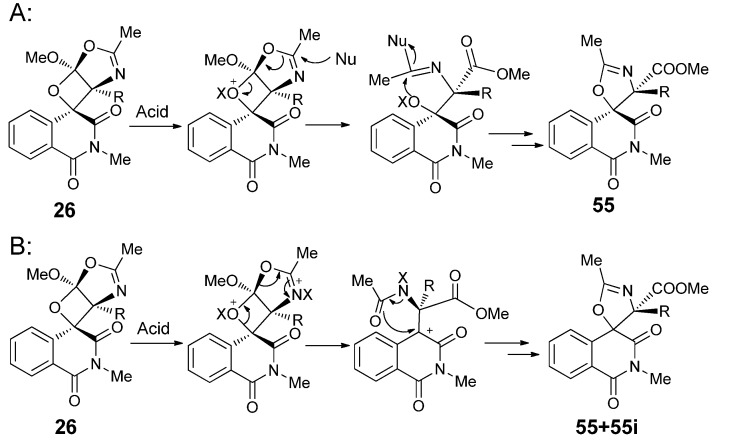
Proposed pathway for the formation of **55** and **55i**.

Reaction of **26** catalyzed by different types and amounts of acids proceeded simultaneously and competitively via pathways A and B, which resulted in different ratios of **55** and **55i** in the ﬁnal products (Scheme 22). Meanwhile, the diastereoselectivity of the transformation was highly dependent on the group R. Bulky substituents like benzoyl were apt to form spiroisoquinolineoxazolines with single configuration, while small ones like methyl led to spiroisoquinolineoxazolines with isomers. Based on the photocycloaddition of **IQT** with 5-methoxyoxazoles and this novel acid-catalyzed transformation of the photocycloadducts, facile syntheses of spiroisoquinolines could be realized. For the spiroisoquinolineoxetanes obtained from photocycloaddition of **IQT** with, their acid-mediated transformation reactions were found to be adjustable through the type and amount of acid used.

### 3.2. Transformations of 1,4-Dioxins

**Scheme 23:**
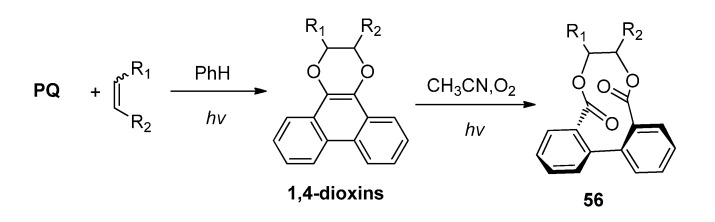
The photo-oxygenations of the [4 + 2] cycloadducts between **PQ** and alkenes.

The [4 + 2] photocycloaddition of some α-diketone such as **PQ** and C=C containing species gave 1,4-dioxins which are relatively stable. Therefore the transformation of 1,4-dioxins has rarely been investigated. Recently we developed a facile photochemical approach to biaryl-containing medium-ring bislactones with easily available reactants via a concise photocycloaddition-photooxidation sequence through the 1,4-dioxin intermediate [25]. Starting from **PQ** or 1,10-phenanthroline-5,6-dione (PN) with C=C-containing compounds, we were able to prepare ten-membered bislactones containing biphenyl or bipyridine in a highly atom-economic way (Scheme 23). The first step in the reaction sequence was the photoinduced [4 + 2] cycloaddition of the dione with the C=C bond to form the dioxinophenanthrene or dioxinophenanthroline. Further transformation of the dioxinophenanthrene or dioxinophenanthroline into the biaryl-containing medium-ring bislactones **56** happened rapidly upon photoirradiation under oxygen atmosphere with yields up to 90%.

## 4. Conclusions

In this paper we have summarized the photo-induced reactions of *α*-diketones with C=C or C≡C- containing compounds with focus on those that have been the subject of our continued interest in the past decade, namely the photoreactions of three type of *α*-diketones: **IS**, **PQ** and **IQT**. The photocycloaddition pathways, including [2 + 2], [4 + 2] and [4 + 4] photocycloadditons, were explained in detail with representative examples from our work and others. The photocycloadditions of **IS** proceed exclusively via a [2 + 2] cycloaddition pathway with simple alkenes or via [4 + 4] cycloaddition pathways with specific oxazole. Complex photocycloadducts have also been obtained from the [4 + 2] photocycloaddition-initiated cascade reactions of **IS**. The photocycloadditions of **PQ** showed stronger competition of the [4 + 2] cycloaddition pathway to the [2 + 2] pathway. Besides, [4 + 4] cycloadducts were also found in the reactions of **PQ** with oxazoles. In contrast, the two carbonyl groups in **IQT** showed distinct reactivity in photocycloadditions. The C-4 carbonyl group is the common reactive site for photocycloadditions and the C-3 carbonyl group seldom gets involved in photoreactions, except in those with special alkenes such as bicyclopropylidene. The pathway partitioning depends not only on the nature of the *α*-diketones, but also on the substituent groups on the C=C or C≡C moieties. Chemo-, regio- and stereoselectivity in the photocycloadditions were also rationalized. Transformations of photocycloadducts were also briefly reviewed, since they could provide facile ways to construct compounds with highly functionalized structures.
